# Assessing the change in prevalence and characteristics of canadians utilizing chiropractic services across two time periods 2001–2010 and 2015–2018: a population-based repeated cross-sectional study

**DOI:** 10.1186/s12998-024-00552-1

**Published:** 2024-10-30

**Authors:** Silvano Mior, Dan Wang, Jessica J. Wong, Sheilah Hogg-Johnson, Pierre Côté

**Affiliations:** 1 Canadian Memorial Chiropractic College, 6100 Leslie Street, Toronto, ON M2H 3J1 Canada; 2 Institute for Disability and Rehabilitation Research, Ontario Tech University, 2000 Simcoe Street North, Oshawa, ON L1H 7K4 Canada; 3 Faculty of Health Sciences, Ontario Tech University, 2000 Simcoe Street North, Oshawa, ON L1H 7K4 Canada; 4 Institute of Health Policy, Management and Evaluation, University of Toronto, 155 College Street, 4th floor, Toronto, ON M5T 3M7 Canada; 5 School of Physical Therapy, Faculty of Health Sciences, Western University, 1201 Western Road, London, ON N6G 1H1 Canada

**Keywords:** Chiropractic, Utilization, Prevalence, Population-based, Musculoskeletal disorders

## Abstract

**Background:**

Despite increases in musculoskeletal disorders (MSD) in Canada, evidence suggests utilization of chiropractic services has remained relatively stable over time. Understanding the extent to which chiropractors are consulted and factors associated with their utilization may suggest factors related to accessing care. We assessed the change in prevalence and characteristics of Canadians seeking chiropractic care across two time periods 2001–2010 and 2015–2018.

**Methods:**

We used national cross-sectional data from seven cycles of the Canadian Community Health Survey between 2001 and 2018. The survey included Canadians aged 12 years and older living in private dwellings in all provinces and territories. National annual weighted prevalence and age-standardized weighted prevalence (and 95% confidence intervals) of chiropractic utilization were calculated. We calculated prevalence of chiropractic utilization stratified by demographic, socioeconomic, lifestyle and health-related variables. Crude linear trends and change in prevalence from 2001 to 2010 were assessed using linear regression models.

**Results:**

The national annual prevalence of Canadians consulting a chiropractor in the previous 12 months slightly increased from 11.0% (95% CI 10.8, 11.3) in 2001 to 11.4% (95%CI 11.1, 11.7) in 2010, and in those reporting receiving regular health care from a chiropractor from 7.5% (95%CI 7.2, 7.7) in 2015 to 7.9% (95%CI 7.7, 8.2) in 2018. Prevalence of utilization varied by province, highest in the Western provinces but lowest in Atlantic provinces. The age-specific prevalence of chiropractic utilization was highest in those aged 35–49 years and remained stable over time, except for slight increase in those aged 65–79 years. A higher percentage of Canadians identifying as white, Canadian-born, in the highest quintile of household income, overweight, physically active and in excellent health reported seeking chiropractic services. The most common reported chronic conditions measured in the survey among Canadians consulting chiropractors were chronic back problems, arthritis, fibromyalgia and headaches.

**Conclusion:**

The national prevalence of utilization of chiropractic services among Canadians slightly increased over time but varied by province and respondents’ socioeconomic and health characteristics. Chronic back problems were the most common reported chronic condition. This comprehensive population-based study on chiropractic utilization in Canada can be used to inform decisions concerning health human resources and access to rehabilitation care for MSD.

## Background

Musculoskeletal disorders, including back pain and arthritis, are leading causes of disability in Canada and globally [1, 2]. Musculoskeletal disorders (MSDs) are also the main reasons for needing rehabilitation; 2.4 billion individuals worldwide have conditions that would benefit from rehabilitation [3]. However, many in need do not receive these services, representing unmet rehabilitation needs that have been substantially increasing in the past 30 years [4]. The most prevalent conditions that would benefit from rehabilitation are MSDs (71%), with low back pain causing the highest burden and the leading condition contributing to unmet rehabilitation needs in Canada and globally [3]. 

MSDs, including back pain, affect approximately one in every five Canadians, and the prevalence of associated disability increases with age [5, 6]. The estimated prevalence of MSDs in Canada has increased from 23.0% in 1990 to 27.8% in 2017 [7]. The 2010 reported costs for MSDs in Canada have been estimated at $8.7 billion, with $6.7 billion ascribed to direct physician, hospital, and medication costs [8]. Overall, MSDs are major contributors to disability and rehabilitation needs, and this burden is projected to increase over time with population growth and aging [1–4]. Given this projected growth, MSDs will place a substantial demand on the health system and health human resources.

Cieza et al. suggest that one strategy to manage such demand is to strengthen rehabilitation services through their integration at the primary care level by improving the training of primary care providers and referral to rehabilitation specialists [3]. In comparison to its high-income peer countries, Canada lags behind in access to regular physicians, timely access to care, formation of interprofessional teams, and communication across the health system, thereby raising concern that not only is primary care in crisis [9], but so is the health workforce in Canada [10]. These concerns are unlikely to dissipate, particularly since adults with MSDs access various health human resources, most commonly physicians, accounting for high rates of visits that further strain health care resources [11, 12]. Shifting primary care physicians’ tasks to other health providers could potentially increase capacity and timely access to non-urgent care, including referral to rehabilitation specialists [3, 13]. Non-medical physician healthcare providers specializing in rehabilitation, including chiropractors, are commonly consulted for MSDs [14]. Referral rates to chiropractors vary but have been reported to be as high as 40% for chronic pain and back problems [15]. 

Chiropractors in Canada are regulated health professionals that provide assessment and treatment of predominantly musculoskeletal conditions. Coverage for their services includes extended health care (EHC), out-of-pocket pay, and provincial and federal public funding, with public funding varying by province [15]. The most common reason for seeking chiropractic care is for MSDs, primarily back pain, neck pain, and extremity problems [14–16]. In 2000/2001, Lim et al. reported that 26% of adults with back pain consulted chiropractors in Canada, compared to 9% among those with no back pain [17], which is similar to the 24% in 2009/2010 [12]. Canizares et al. examined changes in utilization of chiropractic services over time and by birth cohorts from 1994 to 2011 and reported differences in chiropractic utilization by birth cohort but relatively stable national utilization in Canada [18]. Previous studies have assessed a range of variables perceived to impact the utilization of chiropractic services, either at a point in time [12, 17], or over time [18], but are now dated nor did they consider year over year changes in trend.

Recently, Wong et al. assessed the prevalence of healthcare utilization by Canadians with chronic back pain [19]. Using national survey data, they reported that chiropractors were the second most consulted health profession after physicians between 2001 and 2010 and 2015/16 at 24% and 14.5%, respectively. They also reported that those of lower socio-economic status, being an immigrant, older than 65 years, and in fair/poor health were less likely to consult a chiropractor. However, their findings focused specifically on surveyed Canadians with chronic back pain.

Therefore, an up-to-date and comprehensive population-based perspective on chiropractic utilization in Canada is needed to inform decision-making concerning access to rehabilitation care across diverse communities. Understanding the extent to which chiropractors are seen, and factors associated with their utilization, may provide insight to the barriers to accessing care, including in potentially underserved populations in Canada. Elucidating chiropractic utilization and access across sociodemographic factors can inform future tailored strategies to strengthen rehabilitation delivery in Canada.

Hence, a nationwide, comprehensive view on chiropractic utilization among Canadians provides important information to knowledge users, including government and health professional associations, to guide delivery and planning of rehabilitation services provided by chiropractors. We aimed to assess the change in the prevalence and characteristics of Canadians seeking chiropractic care across two time periods 2001–2010 and 2015–2018.

## Methods

Ethical approval for this study was granted by the Research Ethics Board at Ontario Tech University (REB #: 15791–130103). Study results are presented according to the statement described in the Strengthening the Reporting of Observational Studies in Epidemiology (STROBE) Statement [20]. 

### Study population

The present study analyzed national cross-sectional data from seven cycles of the Canadian Community Health Survey (CCHS) in 2001–2018. Conducted by Statistics Canada, CCHS aims to collect health status, health care utilization and health determinants data on annual and nationally representative samples of Canadians aged 12 years and older living in private dwellings in all provinces and territories [21]. Excluded from the survey are persons living on reserves and other Aboriginal settlements, full-time members of the Canadian Forces, those that are institutionalized, children aged 12–17 years that are living in foster care, and persons living in the Quebec health regions of Nunavik and Terres-Cries-de-la-Baie-James.

The sample design, described in detail elsewhere [21], is based on a complex survey design employing a multistage stratified cluster-sampling strategy. This sampling strategy allowed CCHS data to represent approximately 98% of the Canadian population aged 12 years and older living in private dwellings in all provinces and territories, and ensured representativeness of its data at national, provincial, and health region levels, thus providing a more comprehensive and representative picture of health across Canada. As a result, all CCHS cycles cover approximately 97% of the target population who live in the 10 provinces, 94% of those who live in the Yukon, 96% in the Northwest Territories and 93% in Nunavut [21]. In the present study, the seven eligible CCHS cycles were those with national data on chiropractic care utilization. All participants from these seven CCHS cycles were included, and each data cycle was analyzed independently.

### Public involvement

We involved the public, namely an Advisory Committee comprised of executive officers of national and provincial chiropractic associations, in the conduct of our research. The Advisory Committee provided input on selected variables, relevance of findings and readability of reports; however, they were not involved in finalizing the design, analysis nor interpretation of results. Preliminary results were disseminated through presentations at research seminars and scientific meetings for feedback and comprehensibility.

### Equity, diversity and inclusion statement

Our study team included women and men with diverse cultural and ethnic backgrounds, as well as different career levels.

### Data collection

Data were collected by Statistics Canada directly during in-person or telephone computer-assisted interviews [21, 22]. Trained Statistics Canada representatives conducted interviews using standardized questionnaires. The content of the CCHS questionnaire can vary from year to year and includes core and optional modules. Questions included in the core module are consistent across provinces and territories. Provinces and territories can elect to add optional modules to the annual survey of their populations.

### Assessment of utilization of chiropractic services

For five data cycles in 2001–2010, we used the core module of *“Health Care Utilization”* to assess the utilization of chiropractic care services (Yes/No), by asking *“(Not counting when you were an overnight patient) In the past 12 months*,* have you seen or talked to a chiropractor about your physical*,* emotional*,* or mental health? (including both face-to-face and telephone contacts)”*. For 2011–2014, *“Health Care Utilization”* was an optional module for a few provinces, thus we excluded these cycles from our national analysis. For two cycles in 2015-16 (2015) and 2017-18 (2018), the *“Health Care Utilization”* module was replaced by a new core module of *“Primary Health Care”*, which asked participants the following question: *“Other than from your family physician/specialist/nurse practitioner/regular health care provider*,* who do you receive regular health care from?”* [21]. Discrete response options included a range of health care providers, and we defined regular utilization of chiropractic service(yes/no) as reporting receiving regular health care from a chiropractor [21]. All participants in these seven CCHS cycles were eligible for our analysis since all participants from 13 provinces/territories were surveyed for the module of *“Health Care Utilization”* (2001–2010) *or “Primary Health Care*” (2015–2018) allowing us to assess the utilization of chiropractic services.

### Assessment of personal characteristics

We used variables from the eligible seven CCHS cycles between 2001 and 2018 to assess a range of personal characteristics, upon which we conducted our stratified analyses. For variables that were categorized differently in different cycles, regrouping was applied following the *User Guide* provided by Statistics Canada [21] to ensure the consistent categories of all personal characteristics across seven data cycles. When comparable categories between earlier cycles (2001–2010) and later cycles (2015–2018) could not be obtained (i.e., working status last week), we retained the original categorization within each time period and reported the results with cautious interpretation.

We selected variables based upon economic theory related to the use of health care services [17], as well as selected explanatory variables perceived to influence demand for health care as described in the literature [23]. Thus, in the present study, utilization was theorized as a function of discrete variables, namely demographic, socioeconomic, and lifestyle- and health-related characteristics.

The demographic characteristics included age (12–19, 20–34, 35–49, 50–64, 65–79 and 80 + years of age), sex (female, male), province of residence (13 provinces/territories), cultural or racial origin (white, non-white including aboriginal and visible minority), immigrant status (yes, no), length in Canada since immigration (0–9, 10 + years).

Socioeconomic characteristics included education level (less than secondary school graduation, secondary school graduation, some post-secondary education, post-secondary/university degree), household income (1st, 2nd, 3rd, 4th, 5th quintile in national distribution), working status last week (worked, absent, did not have a job, unable/permanent), marital status (married, common-law, widowed/divorced/separated, single).

Lifestyle-related characteristics included type of smoker (daily, occasionally, not at all), type of drinker (regular, occasional, former drinker/did not drink in the past 12 months), and physical activity index (active, moderately active, and inactive).

Health-related characteristics included BMI (underweight, normal, overweight including obese), self-perceived general health (poor, fair, good, very good, excellent), and chronic health conditions (yes, no). Specifically, we used a series of survey questions to independently assess the suffering (yes, no) of 13 chronic conditions that were diagnosed by a health professional and that are expected to last or have lasted 6 months or more, namely back problems, arthritis, fibromyalgia, asthma, migraine headache, diabetes, high blood pressure, heart disease, stroke, cancer, intestinal/stomach ulcers, mood disorder, and anxiety disorder.

### Statistical analysis

To ensure that our final estimates were precise and representative of the Canadian population, we used the survey analysis procedure accounting for survey weights and bootstrap weights provided by Statistics Canada [21]. Survey weights accounted for the multistage sampling and included adjustments for nonresponse and poststratification [21]. Bootstrap weights particularly accounted for the clustering of the samples that allowed for derivation of precise variance estimates [21]. 

For each of the seven CCHS cycles, we computed annual weighted prevalence (and 95% Confidence Interval (CI)) of the utilization of chiropractic services (2001–2010) or receiving regular health care from a chiropractor (2015–2018). We also calculated the weighted prevalence of chiropractic utilization from 2001 to 2018 stratified by the stated characteristics. In all computations, the numerator was the weighted number of individuals in a certain population who reported the utilization of chiropractic services. The denominator was the weighted number of all participants in the study population surveyed for the chiropractic utilization question. With the entire population, we calculated the age-standardized prevalence of chiropractic utilization using the 2015 Canada population [22]. For each data cycle, we used 500 bootstrap weights produced by Statistics Canada and the Bootvar program (version 32) developed by Statistics Canada to compute the 95% CI of prevalence. Bootstrap weights adjust for the complexities of the sampling design, enhancing the precision of variance estimation, which leads to more accurate standard errors, confidence intervals, and hypothesis tests. Bootstrap weights are used to evaluate the quality of survey estimates, and their application is necessary for drawing accurate population-level inferences.

Across the five CCHS cycles in 2001–2010, we used linear regression models to evaluate the crude linear trends in the prevalence of chiropractic utilization and estimated the change in prevalence (regression coefficient β) for every 2 year change, presenting an average change in percentage for every 2 years. The trend was examined in the entire population and by the 29 personal characteristics separately. All statistical tests were 2-sided with a *P* value less than 0.05 considered statistically significant. We report 95% CIs for prevalence estimates and regression coefficients throughout. We used SAS version 9.4 to carry out all the statistical analysis. (Copyright © 2012–2018, SAS Institute Inc., Cary, NC, USA. SAS and all other SAS Institute Inc. product or service names are registered trademarks or trademarks of SAS Institute Inc., Cary, NC, USA.)

## Results

The number of Canadians aged 12 years and older who were sampled in each of the seven cycles of the CCHS from 2001 to 2018 ranged between 110,095 and 135,573. This represented a weighted sample ranging from 25,801,720 to 31,286,303 of the estimated Canadian population in the applicable year (see Table 1).

**Table 1:** Sample size of Canadian Community Health Survey (CCHS), seven cycles from 2001 to 2018

CCHS cycle	2001	2003	2005	2007–2008	2009–2010	2015–2016	2017–2018
Total number of respondents	131,535	135,573	132,947	131,959	124,870	110,095	113,735
Total weighted population	25,801,720	26,578,128	27,131,963	28,030,943	28,737,123	30,602,705	31,286,303

The age-standardized annual weighted prevalence of Canadians who reported consulting a chiropractor in the previous 12 months ranged from 11.0% (95% CI 10.8, 11.3) in 2001 to 11.4% (95%CI 11.1–11.7) in 2010. The national annual prevalence illustrated a small but increasing trend, with an estimated increase of 0.08% (95%CI 0.04–0.11) every two years from 2001 to 2010 (see Table 2). There was also a small increase in the age-standardized prevalence of Canadians who reported receiving regular health care from a chiropractor from 7.5% (95%CI 7.2, 7.7) in 2015 to 7.9% (95%CI 7.7, 8.2) in 2018 (see Table 3).

**Table 2:** National annual prevalence of consulting a chiropractor in the past 12 months, among canadians aged 12 years and older, from 2001 to 2010

CCHS Cycle	2001	2003	2005	2007–2008	2009–2010
N	15,855	15,560	15,387	15,882	14,958
Weighted N	2,879,649	3,002,366	3,072,689	3,189,974	3,306,992
Weighted Prevalence % (95% CI)	11.2 (10.9 to 11.4)	11.3 (11.0 to 11.6)	11.3 (11.1 to 11.6)	11.4 (11.1 to 11.6)	11.5 (11.2 to 11.8)
Age-standardized Weighted Prevalence % (95% CI) *	11.0 (10.8 to 11.3)	11.1 (10.8 to 11.4)	11.2 (10.9 to 11.4)	11.2 (11.0 to 11.5)	11.4 (11.1 to 11.7)

* Age-standardized prevalence was calculated by the direct method using the 2015 Canada population

**Table 3:** National annual prevalence of receiving regular health care from a chiropractor, among canadians aged 12 years and older, from 2015 to 2018

CCHS Cycle	2015–2016	2017–2018
N	8905	9507
Weighted N	2,288,024	2,485,336
Weighted Prevalence % (95% CI)	7.5 (7.2 to 7.7)	7.9 (7.7 to 8.2)
Age-standardized Weighted Prevalence % (95% CI) *	7.5 (7.2 to 7.7)	7.9 (7.7 to 8.2)

* Age-standardized prevalence was calculated by the direct method using the 2015 Canada population

### Prevalence of chiropractic utilization stratified by demographic characteristics

The demographic characteristics of Canadians who reported consulting a chiropractor from 2001 to 2010 are summarized in Table 4. The prevalence of consulting a chiropractor from 2001 to 2010 was highest in the age group 35–49 and lowest for those over 80 years of age. This prevalence was stable over time for all age groups except those ages 65–79 years, where the average prevalence increased by 0.16% (95%CI 0.09, 0.23) every two years. Males and females showed similar prevalence of consulting a chiropractor, with a stable trend during this period. Compared to 2001–2010, a similar age-specific distribution in prevalence of Canadians reporting receiving regular health care from chiropractors was observed in the 2015 and 2018 cycles (Table 5), with one notable exception: prevalence in the age group 65–79 slightly increased over time. Moreover, there was a small increase in prevalence among those aged 20–34 from 2015 to 2018 reporting receiving regular health care from a chiropractor (Table 5).

**Table 4:** National annual prevalence(95%CI) of canadians who consulted a chiropractor about their physical, emotional, or mental health, stratified by personal characteristics, CCHS 2001–2010

	Weighted % (95%CI)					
	2001–2002	2003–2004	2005–2006	2007–2008	2009–2010	β (95% CI) ^a^
**All (weighted prevalence)**	11.2 (10.9 to 11.4)	11.3 (11.0 to 11.6)	11.3 (11.1 to 11.6)	11.4 (11.1 to 11.6)	11.5 (11.2 to 11.8)	0.08 (0.04 to 0.11)
**Demographic characteristics**						
**Age group (year)**						
12–19	7.8 (7.3 to 8.4)	8.1 (7.5 to 8.7)	8.3 (7.7 to 8.9)	7.5 (7.0 to 8.1)	8.3 (7.5 to 9.0)	0.03 (-0.33 to 0.38)
20–34	11.0 (10.4 to 11.5)	11.2 (10.6 to 11.8)	11.1 (10.6 to 11.6)	11.2 (10.7 to 11.8)	10.9 (10.3 to 11.4)	-0.02 (-0.20 to 0.15)
35–49	13.3 (12.8 to 13.8)	13.8 (13.2 to 14.4)	13.5 (12.9 to 14.1)	14.1 (13.5 to 14.7)	14.0 (13.3 to 14.7)	0.17 (-0.07 to 0.41)
50–64	12.5 (11.9 to 13.1)	12.0 (11.5 to 12.6)	12.5 (12.0 to 13.1)	12.4 (11.9 to 13.0)	13.0 (12.3 to 13.6)	0.14 (-0.16 to 0.43)
65–79	8.7 (8.2 to 9.3)	8.8 (8.2 to 9.3)	9.0 (8.5 to 9.5)	9.1 (8.6 to 9.7)	9.4 (8.8 to 9.9)	0.16 (0.09 to 0.23)
80+	5.9 (5.0 to 6.7)	5.9 (5.2 to 6.7)	6.3 (5.6 to 7.1)	5.4 (4.8 to 6.1)	6.1 (5.2 to 6.9)	-0.01 (-0.38 to 0.36)
**Sex**						
Male	10.9 (10.5 to 11.2)	11.0 (10.6 to 11.4)	11.2 (10.8 to 11.6)	10.8 (10.4 to 11.1)	11.0 (10.6 to 11.4)	0.00 (-0.18 to 0.19)
Female	11.4 (11.1 to 11.8)	11.6 (11.2 to 12.0)	11.4 (11.1 to 11.8)	12.0 (11.6 to 12.3)	12.0 (11.6 to 12.4)	0.15 (-0.02 to 0.32)
**Province of residence**						
Newfoundland & Labrador	3.2 (2.5 to 3.9)	4.4 (3.0 to 5.7)	4.0 (3.2 to 4.8)	4.4 (3.6 to 5.2)	5.4 (4.4 to 6.4)	0.43 (0.01 to 0.86)
Prince Edward Island	3.6 (2.8 to 4.4)	4.2 (3.0 to 5.5)	3.3 (2.2 to 4.3)	2.9 (2.1 to 3.7)	3.6 (2.5 to 4.6)	-0.14 (-0.66 to 0.38)
Nova Scotia	3.2 (2.6 to 3.9)	3.9 (3.1 to 4.6)	5.8 (4.8 to 6.9)	5.5 (4.7 to 6.3)	6.5 (5.5 to 7.4)	0.80 (0.24 to 1.36)
New Brunswick	3.9 (3.3 to 4.6)	4.6 (3.9 to 5.4)	5.2 (4.4 to 6.1)	6.1 (5.3 to 6.9)	5.8 (4.8 to 6.7)	0.51 (0.13 to 0.90)
Quebec	8.8 (8.3 to 9.4)	8.8 (8.2 to 9.4)	8.9 (8.4 to 9.4)	8.1 (7.7 to 8.6)	8.6 (8.1 to 9.2)	-0.10 (-0.40 to 0.20)
Ontario	10.7 (10.3 to 11.2)	11.2 (10.8 to 11.7)	10.7 (10.3 to 11.1)	10.9 (10.5 to 11.4)	11.2 (10.7 to 11.7)	0.06 (-0.21 to 0.34)
Manitoba	17.6 (16.3 to 18.8)	17.5 (16.1 to 18.9)	17.5 (16.2 to 18.8)	17.5 (16.2 to 18.8)	18.9 (17.3 to 20.6)	0.27 (-0.26 to 0.81)
Saskatchewan	13.7 (12.7 to 14.8)	15.2 (14.0 to 16.4)	15.7 (14.5 to 16.9)	16.9 (15.7 to 18.0)	16.4 (15.0 to 17.8)	0.69 (0.09 to 1.30)
Alberta	17.1 (16.3 to 18.0)	17.7 (16.7 to 18.7)	17.5 (16.4 to 18.5)	17.7 (16.6 to 18.8)	17.1 (16.0 to 18.2)	-0.01 (-0.36 to 0.34)
British Columbia	14.6 (13.9 to 15.2)	12.9 (12.1 to 13.7)	14.0 (13.2 to 14.7)	14.2 (13.4 to 15.0)	13.2 (12.3 to 14.0)	-0.15 (-0.92 to 0.62)
Yukon/Northwest Territories/Nunavut	7.4 (6.3 to 8.5)	7.2 (5.8 to 8.5)	7.2 (5.8 to 8.6)	5.3 (4.1 to 6.5)	5.3 (4.3 to 6.2)	-0.62 (-1.19 to -0.04)
**Cultural / racial origin**						
White	12.1 (11.8 to 12.3)	12.3 (12.0 to 12.6)	12.4 (12.2 to 12.7)	12.6 (12.3 to 12.9)	12.7 (12.4 to 13.1)	0.17 (0.13 to 0.20)
Non-white (Indigenous/Visible Minority)	5.8 (5.2 to 6.5)	6.2 (5.6 to 6.8)	6.4 (5.8 to 6.9)	6.6 (6.0 to 7.2)	7.1 (6.4 to 7.7)	0.29 (0.20 to 0.37)
**Immigrant status**						
Landed immigrant / non-permanent resident	7.4 (6.8 to 7.9)	7.5 (6.9 to 8.1)	7.9 (7.3 to 8.4)	7.9 (7.4 to 8.4)	8.5 (7.8 to 9.2)	0.28 (0.13 to 0.43)
Canadian born	12.2 (11.9 to 12.4)	12.4 (12.1 to 12.7)	12.4 (12.1 to 12.7)	12.5 (12.1 to 12.8)	12.5 (12.2 to 12.8)	0.08 (0.02 to 0.13)
**Length/time in Canada since immigration**						
0–9 years	5.2 (4.2 to 6.1)	4.5 (3.5 to 5.6)	5.1 (4.0 to 6.2)	5.9 (4.9 to 6.8)		0.27 (-0.70 to 1.24)
≥ 10 years	8.2 (7.6 to 8.9)	8.5 (7.8 to 9.3)	8.9 (8.2 to 9.6)	8.8 (8.1 to 9.4)	9.6 (8.8 to 10.5)	0.31 (0.06 to 0.56)
**Socioeconomic characteristics**						
**Highest education level**						
Less than secondary school graduation	8.5 (8.1 to 8.9)	8.3 (7.9 to 8.7)	8.1 (7.7 to 8.5)	7.9 (7.5 to 8.3)	8.0 (7.5 to 8.6)	-0.13 (-0.26 to -0.00)
Secondary school graduation, no post- secondary education	11.4 (10.8 to 12.0)	11.8 (11.2 to 12.4)	11.6 (10.9 to 12.3)	11.0 (10.4 to 11.6)	11.5 (10.8 to 12.3)	-0.05 (-0.38 to 0.27)
Some post-secondary education	13.2 (12.2 to 14.2)	12.1 (11.1 to 13.2)	11.2 (10.4 to 12.1)	11.3 (10.4 to 12.3)	11.8 (10.8 to 12.8)	-0.36 (-1.01 to 0.28)
Post-secondary certificate/diploma or university degree	12.5 (12.1 to 12.9)	12.7 (12.3 to 13.2)	13.0 (12.6 to 13.4)	13.1 (12.7 to 13.5)	13.0 (12.5 to 13.4)	0.13 (-0.00 to 0.26)
**Household income**						
1st quintile (lowest)	8.0 (6.9 to 9.1)	7.8 (6.4 to 9.2)	6.8 (6.3 to 7.3)	6.9 (6.3 to 7.4)	6.8 (6.2 to 7.4)	-0.32 (-0.66 to 0.02)
2nd quintile	6.8 (6.1 to 7.5)	6.9 (6.1 to 7.7)	10.4 (9.8 to 11.0)	9.4 (8.8 to 10.0)	10.5 (9.7 to 11.3)	0.98 (-0.15 to 2.11)
3rd quintile	9.5 (8.9 to 10.0)	9.5 (8.9 to 10.1)	12.1 (11.5 to 12.7)	13.0 (12.4 to 13.7)	12.8 (12.0 to 13.5)	1.02 (0.16 to 1.87)
4th quintile	12.0 (11.5 to 12.4)	11.9 (11.4 to 12.3)	13.5 (12.8 to 14.2)	13.9 (13.2 to 14.6)	14.6 (13.8 to 15.4)	0.74 (0.32 to 1.16)
5th quintile (highest)	13.3 (12.8 to 13.8)	13.7 (13.1 to 14.2)	14.7 (14.0 to 15.4)	15.6 (14.9 to 16.4)	15.2 (14.4 to 15.9)	0.56 (0.09 to 1.04)
**Working status last week (age 15–75)**						
Worked at a job / business	12.8 (12.5 to 13.2)	13.2 (12.7 to 13.6)	13.0 (12.7 to 13.4)	13.1 (12.7 to 13.5)	13.3 (12.9 to 13.7)	0.09 (-0.02 to 0.21)
Absent from work / business	14.1 (12.8 to 15.5)	13.9 (12.6 to 15.3)	14.0 (12.8 to 15.2)	14.7 (13.4 to 16.0)	15.5 (13.9 to 17.2)	0.36 (-0.06 to 0.78)
Did not have a job	9.1 (8.7 to 9.5)	9.0 (8.6 to 9.5)	9.4 (8.9 to 9.8)	9.2 (8.7 to 9.6)	9.2 (8.7 to 9.7)	0.04 (-0.09 to 0.17)
Unable/permanent	8.2 (6.7 to 9.7)	8.0 (6.6 to 9.3)	7.9 (6.5 to 9.4)	8.6 (7.1 to 10.1)	8.3 (6.6 to 9.9)	0.07 (-0.20 to 0.34)
**Marital status**						
Married	12.4 (12.0 to 12.7)	12.4 (11.9 to 12.8)	12.8 (12.4 to 13.2)	13.0 (12.6 to 13.4)	13.4 (12.9 to 13.9)	0.27 (0.14 to 0.40)
Common-law	12.3 (11.4 to 13.2)	13.4 (12.3 to 14.5)	12.3 (11.4 to 13.1)	13.3 (12.4 to 14.3)	11.8 (10.9 to 12.8)	-0.10 (-0.90 to 0.69)
Widowed/Divorced/Separated	10.8 (10.2 to 11.4)	11.0 (10.3 to 11.6)	10.4 (9.8 to 11.1)	10.1 (9.4 to 10.7)	10.0 (9.3 to 10.8)	-0.23 (-0.45 to -0.02)
Single	9.0 (8.6 to 9.4)	9.1 (8.6 to 9.5)	8.9 (8.5 to 9.3)	8.7 (8.3 to 9.1)	8.9 (8.5 to 9.4)	-0.05 (-0.19 to 0.09)
**Lifestyle-related characteristics**						
**Type of smoker**						
Daily	9.8 (9.4 to 10.3)	10.0 (9.5 to 10.6)	9.6 (9.0 to 10.2)	9.0 (8.5 to 9.6)	9.1 (8.5 to 9.8)	-0.23 (-0.49 to 0.02)
Occasionally	11.7 (10.5 to 12.9)	12.0 (10.6 to 13.3)	10.5 (9.4 to 11.6)	11.8 (10.5 to 13.2)	11.2 (9.8 to 12.6)	-0.11 (-0.77 to 0.55)
Not at all	11.5 (11.2 to 11.8)	11.5 (11.2 to 11.9)	11.8 (11.5 to 12.1)	11.9 (11.6 to 12.2)	12.0 (11.7 to 12.3)	0.12 (0.07 to 0.17)
**Type of drinker**						
Regular drinker	12.5 (12.1 to 12.8)	12.5 (12.1 to 12.8)	12.7 (12.3 to 13.0)	12.8 (12.5 to 13.2)	13.0 (12.7 to 13.4)	0.16 (0.09 to 0.22)
Occasional drinker	10.3 (9.8 to 10.8)	11.2 (10.6 to 11.8)	10.7 (10.2 to 11.3)	11.0 (10.4 to 11.7)	11.2 (10.5 to 11.9)	0.15 (-0.19 to 0.49)
Did not drink in the last 12 months (including former drinker)	8.7 (8.2 to 9.2)	8.4 (7.9 to 8.9)	8.3 (7.9 to 8.8)	7.9 (7.4 to 8.3)	7.9 (7.3 to 8.4)	-0.23 (-0.34 to -0.11)
**Physical activity index**						
Active	12.5 (12.0 to 13.1)	12.6 (12.1 to 13.2)	11.7 (11.2 to 12.2)	12.7 (12.2 to 13.2)	12.8 (12.2 to 13.4)	0.06 (-0.43 to 0.55)
Moderate active	12.3 (11.8 to 12.8)	11.7 (11.1 to 12.2)	12.6 (12.0 to 13.1)	12.0 (11.5 to 12.5)	11.9 (11.3 to 12.6)	-0.04 (-0.44 to 0.36)
Inactive	10.4 (10.0 to 10.7)	10.7 (10.3 to 11.1)	10.7 (10.3 to 11.1)	10.6 (10.3 to 11.0)	10.8 (10.3 to 11.2)	0.08 (-0.03 to 0.18)
**Health-related characteristics**						
**BMI (aged ≥ 18 years)**						
Underweight	9.6 (8.5 to 10.6)	8.0 (6.7 to 9.3)	8.2 (6.8 to 9.6)	7.7 (6.2 to 9.2)	8.0 (6.1 to 10.0)	-0.34 (-0.93 to 0.24)
Normal weight	12.1 (11.6 to 12.6)	11.3 (10.9 to 11.7)	11.2 (10.8 to 11.6)	11.3 (10.9 to 11.7)	11.3 (10.8 to 11.8)	-0.15 (-0.49 to 0.18)
Overweight (including obese)	10.8 (10.5 to 11.1)	12.3 (11.9 to 12.7)	12.4 (12.0 to 12.8)	12.8 (12.4 to 13.2)	12.7 (12.2 to 13.2)	0.43 (-0.07 to 0.92)
**Self-perceived general health**						
Poor	10.7 (9.2 to 12.1)	8.8 (7.5 to 10.0)	8.6 (7.3 to 9.8)	9.1 (7.8 to 10.5)	9.3 (7.7 to 10.9)	-0.24 (-1.09 to 0.62)
Fair	10.5 (9.8 to 11.2)	10.2 (9.4 to 11.0)	10.3 (9.6 to 11.1)	10.2 (9.4 to 11.1)	11.2 (10.2 to 12.3)	0.15 (-0.50 to 0.79)
Good	11.5 (11.1 to 12.0)	11.0 (10.5 to 11.4)	11.4 (10.9 to 11.9)	11.0 (10.5 to 11.5)	11.4 (10.9 to 11.9)	-0.02 (-0.31 to 0.26)
Very good	11.6 (11.2 to 12.0)	12.0 (11.5 to 12.5)	12.1 (11.7 to 12.5)	12.1 (11.7 to 12.5)	12.0 (11.5 to 12.5)	0.10 (-0.06 to 0.26)
Excellent	10.5 (10.0 to 10.9)	11.3 (10.8 to 11.9)	10.7 (10.1 to 11.3)	11.4 (10.8 to 12.0)	11.1 (10.5 to 11.8)	0.14 (-0.26 to 0.54)
**Has chronic health condition**						
Yes	13.3 (13.0 to 13.6)	13.1 (12.7 to 13.4)	13.1 (12.8 to 13.4)	/	/	-0.11 (-1.17 to 0.96)
No	7.4 (7.0 to 7.7)	7.5 (7.1 to 7.8)	7.5 (7.1 to 7.9)	/	/	0.05 (-0.17 to 0.27)
**Back problem**						
Yes	25.6 (24.9 to 26.4)	24.0 (23.2 to 24.8)	24.0 (23.2 to 24.8)	22.8 (22.0 to 23.5)	23.6 (22.7 to 24.5)	-0.53 (-1.25 to 0.20)
No	8.1 (7.8 to 8.3)	8.2 (7.9 to 8.4)	8.4 (8.1 to 8.6)	8.5 (8.3 to 8.7)	8.7 (8.4 to 9.0)	0.16 (0.13 to 0.19)
**Arthritis**						
Yes	13.4 (12.8 to 13.9)	12.5 (11.9 to 13.1)	13.2 (12.6 to 13.8)	13.1 (12.4 to 13.7)	13.1 (12.3 to 13.8)	0.00 (-0.36 to 0.37)
No	10.8 (10.5 to 11.0)	11.0 (10.7 to 11.3)	11.0 (10.7 to 11.3)	11.1 (10.8 to 11.4)	11.4 (11.1 to 11.7)	0.14 (0.02 to 0.25)
**Fibromyalgia**						
Yes	21.6 (18.9 to 24.4)	18.5 (16.1 to 20.8)	19.9 (17.3 to 22.4)	/	/	-0.87 (-17.7 to 15.90)
No	11.0 (10.8 to 11.3)	11.2 (10.9 to 11.5)	11.2 (10.9 to 11.5)	/	/	0.08 (-0.38 to 0.54)
**Asthma**						
Yes	12.3 (11.4 to 13.1)	13.5 (12.5 to 14.6)	13.4 (12.4 to 14.3)	12.4 (11.5 to 13.3)	12.1 (11.1 to 13.2)	-0.13 (-0.84 to 0.58)
No	11.1 (10.8 to 11.3)	11.1 (10.8 to 11.4)	11.1 (10.9 to 11.4)	11.3 (11.0 to 11.6)	11.4 (11.1 to 11.8)	0.10 (0.04 to 0.16)
**Headache**						
Yes	15.7 (14.7 to 16.6)	14.4 (13.5 to 15.4)	15.4 (14.5 to 16.4)	14.5 (13.5 to 15.4)	15.9 (14.8 to 17.0)	0.05 (-0.76 to 0.85)
No	10.7 (10.5 to 11.0)	10.9 (10.6 to 11.2)	10.8 (10.6 to 11.1)	11.0 (10.8 to 11.3)	11.0 (10.7 to 11.3)	0.07 (-0.01 to 0.15)
**Diabetes**						
Yes	9.2 (8.2 to 10.2)	9.5 (8.3 to 10.7)	9.5 (8.5 to 10.5)	9.5 (8.5 to 10.5)	9.1 (8.2 to 10.1)	-0.02 (-0.23 to 0.20)
No	11.2 (11.0 to 11.5)	11.4 (11.1 to 11.7)	11.4 (11.2 to 11.7)	11.5 (11.2 to 11.8)	11.7 (11.4 to 12.0)	0.10 (0.06 to 0.13)
**High blood pressure**						
Yes	9.6 (9.0 to 10.2)	9.5 (8.9 to 10.1)	10.2 (9.6 to 10.8)	10.3 (9.7 to 10.9)	10.1 (9.5 to 10.8)	0.19 (-0.07 to 0.46)
No	11.4 (11.1 to 11.7)	11.6 (11.3 to 11.9)	11.5 (11.2 to 11.8)	11.6 (11.3 to 11.9)	11.8 (11.5 to 12.1)	0.08 (-0.01 to 0.16)
**Heart disease**						
Yes	8.8 (7.9 to 9.7)	9.1 (8.1 to 10.0)	8.9 (7.9 to 9.9)	8.2 (7.3 to 9.0)	8.6 (7.6 to 9.6)	-0.14 (-0.46 to 0.18)
No	11.3 (11.0 to 11.5)	11.4 (11.1 to 11.7)	11.4 (11.2 to 11.7)	11.6 (11.3 to 11.8)	11.7 (11.4 to 12.0)	0.09 (0.07 to 0.11)
**Stroke**						
Yes	7.7 (6.0 to 9.4)	8.1 (5.8 to 10.5)	7.2 (5.6 to 8.9)	8.2 (6.4 to 10.0)	6.7 (5.1 to 8.3)	-0.19 (-0.84 to 0.45)
No	11.2 (10.9 to 11.4)	11.3 (11.1 to 11.6)	11.4 (11.1 to 11.6)	11.4 (11.2 to 11.7)	11.6 (11.3 to 11.9)	0.08 (0.05 to 0.11)
**Cancer**						
Yes	10.1 (8.5 to 11.7)	9.9 (8.0 to 11.8)	9.5 (7.6 to 11.4)	12.0 (9.9 to 14.0)	10.1 (8.0 to 12.2)	0.21 (-0.85 to 1.26)
No	11.2 (10.9 to 11.4)	11.3 (11.0 to 11.6)	11.3 (11.1 to 11.6)	11.4 (11.1 to 11.6)	11.5 (11.2 to 11.8)	0.08 (0.03 to 0.12)
**Intestinal ulcer**						
Yes	13.7 (12.2 to 15.1)	12.4 (11.0 to 13.8)	13.0 (11.4 to 14.7)	11.9 (10.3 to 13.4)	13.1 (11.2 to 14.9)	-0.17 (-0.92 to 0.58)
No	11.1 (10.8 to 11.3)	11.2 (11.0 to 11.5)	11.3 (11.0 to 11.5)	11.4 (11.1 to 11.6)	11.5 (11.2 to 11.8)	0.09 (0.06 to 0.12)
**Mood disorder**						
Yes	/	14.4 (13.2 to 15.5)	13.5 (12.4 to 14.6)	13.2 (12.1 to 14.2)	13.6 (12.5 to 14.7)	-0.25 (-1.16 to 0.66)
No	/	11.1 (10.9 to 11.4)	11.2 (10.9 to 11.5)	11.3 (11.0 to 11.5)	11.4 (11.1 to 11.7)	0.08 (0.05 to 0.11)
**Anxiety**						
Yes	/	12.9 (11.6 to 14.2)	12.6 (11.4 to 13.8)	11.2 (10.1 to 12.2)	13.5 (12.1 to 14.9)	0.04 (-2.29 to 2.37)
No	/	11.2 (11.0 to 11.5)	11.3 (11.0 to 11.5)	11.4 (11.1 to 11.7)	11.4 (11.1 to 11.7)	0.07 (0.01 to 0.13)

^a^The association estimate β (95% CI) from linear regression model. With the Canadian Community Health Survey 2-year cycle as a continuous variable, the value of B estimate indicates the average percentage point change in prevalence of chiropractic utilization every 2 years

**Table 5:** National annual prevalence(95%CI) of canadians who receive regular health care from chiropractors, stratified by personal characteristics, CCHS 2015–2018

	Weighted % (95%CI)	
	2015–2016	2017–2018
**All**	7.5 (7.2 to 7.7)	7.9 (7.7 to 8.2)
**Demographic characteristics**		
**Age group (year)**		
12–19	5.7 (5.1 to 6.2)	5.8 (5.2 to 6.5)
20–34	5.8 (5.3 to 6.2)	6.9 (6.3 to 7.4)
35–49	9.4 (8.8 to 10.0)	9.2 (8.7 to 9.8)
50–64	8.8 (8.3 to 9.4)	9.8 (9.3 to 10.4)
65–79	7.1 (6.6 to 7.6)	6.9 (6.5 to 7.4)
80+	4.0 (3.4 to 4.6)	4.7 (3.8 to 5.5)
**Sex**		
Male	7.0 (6.7 to 7.4)	7.1 (6.7 to 7.4)
Female	7.9 (7.6 to 8.2)	8.8 (8.4 to 9.1)
**Province of residence**		
Nfld. & Labrador	4.8 (3.7 to 5.8)	4.2 (3.2 to 5.1)
Prince Edward Island	2.7 (1.9 to 3.6)	2.8 (1.8 to 3.8)
Nova Scotia	6.0 (5.1 to 6.9)	5.0 (4.2 to 5.8)
New Brunswick	3.3 (2.4 to 4.2)	3.5 (2.8 to 4.3)
Quebec	4.6 (4.3 to 5.0)	5.2 (4.8 to 5.6)
Ontario	7.8 (7.4 to 8.2)	8.3 (7.8 to 8.8)
Manitoba	11.0 (9.9 to 12.1)	10.1 (9.1 to 11.2)
Saskatchewan	11.6(10.4 to 12.9)	12.9(11.3 to 14.6)
Alberta	11.1(10.3 to 11.9)	12.6(11.7 to 13.4)
British Columbia	8.1 (7.5 to 8.8)	8.0 (7.3 to 8.7)
Yukon/NWT/Nunavut	3.0 (2.2 to 3.7)	3.3 (2.4 to 4.1)
**Cultural / racial origin**		
White	8.8 (8.5 to 9.1)	9.2 (8.9 to 9.5)
Non-white (Aboriginal or Visible Minority)	4.3 (3.9 to 4.7)	4.9 (4.4 to 5.3)
**Immigrant status (D)**		
Landed immigrant / non-permanent resident	4.6 (4.2 to 5.1)	4.8 (4.3 to 5.2)
Non-immigrant (Canadian born)	8.7 (8.4 to 9.0)	9.2 (8.9 to 9.5)
**Length/time in Canada since imm.**		
0–9 years	2.8 (2.1 to 3.5)	3.3 (2.4 to 4.3)
10 + years	5.4 (4.8 to 6.0)	5.6 (5.1 to 6.2)
**Socioeconomic characteristics**		
**Highest education level**		
Less than secondary school graduation	5.3 (4.9 to 5.8)	5.4 (5.0 to 5.8)
Secondary school graduation, no post-secondary education	7.1 (6.6 to 7.6)	7.3 (6.8 to 7.9)
Some post-secondary education	8.4 (8.0 to 8.7)	9.0 (8.7 to 9.4)
**Household income**		
1st quintile	3.5 (3.1 to 3.9)	4.0 (3.5 to 4.4)
2nd quintile	5.7 (5.2 to 6.2)	6.1 (5.6 to 6.6)
3rd quintile	7.8 (7.2 to 8.3)	7.9 (7.3 to 8.5)
4th quintile	9.9 (9.2 to10.5)	10.0 (9.4 to 10.6)
5th quintile	10.7(10.1 to 11.2)	11.8(11.1 to 12.4)
Working status last week* (age 15–75)		
Worked at a job / business	8.6 (8.2 to 8.9)	9.0 (8.7 to 9.4)
Absent from work / business	11.1 (9.6 to 12.5)	10.7 (9.4 to 12.0)
Did not have a job - last week	6.2 (5.8 to 6.6)	6.5 (6.1 to 6.8)
**Marital status - (G)**		
Married	9.2 (8.8 to 9.6)	9.5 (9.1 to 9.9)
Common-law	7.8 (7.1 to 8.6)	8.3 (7.4 to 9.1)
Widowed/Divorced/Separated	6.3 (5.8 to 6.8)	7.4 (6.8 to 7.9)
Single	5.2 (4.9 to 5.5)	5.6 (5.3 to 6.0)
**Lifestyle-related characteristics**		
**Type of smoker**		
Daily	5.1 (4.6 to 5.6)	5.2 (4.6 to 5.8)
Occasionally	6.4 (5.4 to 7.4)	6.9 (5.8 to 8.1)
Not at all	7.9 (7.6 to 8.2)	8.4 (8.1 to 8.6)
**Type of drinker**		
Regular drinker	8.7 (8.4 to 9.1)	9.1 (8.7 to 9.4)
Occasional drinker	6.5 (5.9 to 7.0)	7.4 (6.8 to 8.0)
Did not drink in the last 12 months (including former drinker)	5.0 (4.6 to 5.4)	5.4 (5.0 to 5.8)
**Physical activity index**		
Active	8.5 (8.2 to 8.9)	9.0 (8.6 to 9.4)
Moderate active	7.2 (6.7 to 7.7)	7.8 (7.2 to 8.4)
Inactive	5.6 (5.2 to 6.1)	6.1 (5.6 to 6.5)
**Health-related indicators**		
**BMI (aged 18+)**		
Underweight	5.0 (3.6 to 6.4)	4.7 (3.3 to 6.0)
Normal weight	6.9 (6.5 to 7.3)	7.7 (7.3 to 8.1)
Overweight (including obese)	8.6 (8.2 to 9.0)	8.9 (8.6 to 9.3)
**Self-perceived general health**		
Poor		
Fair	6.9 (6.0 to 7.8)	7.4 (6.6 to 8.2)
Good	7.2 (6.7 to 7.6)	7.5 (7.0 to 7.9)
Very good	7.9 (7.5 to 8.3)	8.8 (8.3 to 9.2)
Excellent	7.6 (7.1 to 8.1)	7.4 (6.9 to 7.9)
**Chronic health condition**		
***Back problem***		
Yes	14.2(13.5 to 15.0)	/
No	5.9 (5.7 to 6.2)	/
***Arthritis***		
Yes	9.4 (8.9 to 10.0)	9.8 (9.1 to 10.4)
No	7.0 (6.7 to 7.3)	7.5 (7.3 to 7.8)
***Fibromyalgia***		
Yes	10.0 (8.5 to 11.6)	/
No	7.4 (7.2 to 7.7)	/
***Asthma***		
Yes	8.9 (7.9 to 9.8)	9.6 (8.7 to 10.6)
No	7.4 (7.1 to 7.6)	7.8 (7.5 to 8.1)
***Headache***		
Yes	9.7 (8.9 to 10.5)	/
No	7.2 (7.0 to 7.5)	/
***Diabetes***		
Yes	6.3 (5.4 to 7.1)	6.4 (5.6 to 7.1)
No	7.6 (7.3 to 7.8)	8.1 (7.8 to 8.3)
***High blood pressure***		
Yes	7.3 (6.8 to 7.8)	7.2 (6.7 to 7.7)
No	7.5 (7.3 to 7.8)	8.1 (7.8 to 8.4)
***Heart disease***		
Yes	6.9 (5.8 to 8.1)	6.1 (5.3 to 6.9)
No	7.5 (7.3 to 7.8)	8.0 (7.8 to 8.3)
***Stroke***		
Yes	5.2 (2.2 to 8.2)	5.5 (4.1 to 6.9)
No	7.5 (7.3 to 7.7)	8.0 (7.7 to 8.2)
***Cancer***		
Yes	7.7 (5.2 to10.3)	6.2 (4.7 to 7.7)
No	7.5 (7.2 to 7.7)	8.0 (7.7 to 8.2)
***Mood disorder***		
Yes	8.6 (7.8 to 9.4)	9.9 (8.9 to11.0)
No	7.4 (7.1 to 7.6)	7.8 (7.5 to 8.0)
***Anxiety***		
Yes	8.3 (7.5 to 9.2)	9.2 (8.3 to 10.0)
No	7.4 (7.2 to 7.6)	7.8 (7.5 to 8.1)

Annual prevalence of Canadians consulting a chiropractor varied by province with the lowest in the Atlantic provinces and highest in the western provinces, specifically in Manitoba and Alberta (Table 4). In five of the 11 jurisdictions sampled, significant change in the annual prevalence was observed from 2001 to 2010, with the highest being in Nova Scotia where the average percentage increase every two years was 0.80% (95%CI 0.24, 1.36) but with a decreasing trend (β = -0.62 (95%CI -1.19, -0.04)) in the territories. A similar pattern was observed in 2015 and 2018 cycles, with the lowest reported prevalence in the Atlantic provinces and territories and highest in the mid-western provinces among Canadians reporting receiving regular health care from a chiropractor (Table 5).

A higher percentage of Canadians identifying as white compared to those identifying as non-white (Aboriginal/visible minority) reported consulting a chiropractor (Table 4). The prevalence in both groups increased from 2001 to 2010, with an average growth of 0.17% (95% CI 0.13, 0.20) and 0.29% (95%CI 0.20, 0.37) every two years, respectively. Similar findings and trends were observed in those identifying as non-immigrant (Canadian born) and landed immigrant. Furthermore, the prevalence of consulting a chiropractor among landed immigrants differed by their length of time in Canada, with higher prevalence and increasing trend among those reporting 10 or more years since immigrating (Table 4). Despite a change in the question wording. i.e., receiving regular health care from a chiropractor, similar patterns were observed in 2015 and 2018 (Table 5).

### Prevalence of chiropractic utilization stratified by socioeconomic characteristics

Canadians with less than secondary school education had the lowest prevalence of consulting a chiropractor with prevalence decreasing over time, as opposed to the increase seen in those with post-secondary education (Table 4). Similarly, those in the lowest quintile of household income had the lowest prevalence with decreasing trend in consulting chiropractors between 2001 and 2010. There was a significant increasing trend in Canadians with higher household income level (the 3rd to 5th quintiles), with highest percentage seen in the 5th quintile and highest increase seen in the 4th quintile (β = 0.74, 95%CI 0.32, 1.16). Working Canadians and those absent from work had the highest prevalence. Higher prevalence was also seen for those reported to be married, with an increasing trend for those who were married whereas there was a decreasing trend for those reporting being widowed/divorced/separated (Table 4). Similar prevalence was reported in the years 2015 and 2018 among those reporting receiving regular health care from a chiropractor (Table 5).

### Prevalence of chiropractic utilization stratified by lifestyle and health-related characteristics

Canadians who reported not being a daily smoker and those who drank regularly showed a higher prevalence of consulting a chiropractor (Table 4). Between 2001 and 2010, there was an increasing trend among non-smokers and regular drinkers, while the trend decreased in those who had not had a drink in the previous 12 months. There is an increasing trend in Canadians who identified being active and inactive, overweight, and in fair, very good and excellent self-perceived general health who reported consulting a chiropractor between 2001 and 2010 (Table 4). The prevalence of Canadians reporting receiving regular health care from a chiropractor in the year 2018 compared to 2015 for all categories was similar except for a higher prevalence in 2018 for those between 20 and 34 age group (Table 5).

Of Canadians reporting consulting a chiropractor in the previous year, almost 24% reported having a chronic back problem, 20% reported fibromyalgia, 16% headaches, 14% a mood disorder, and 13% arthritis or asthma, percentages that remained stable between 2001 and 2010 (Table 4). In 2015, 14.2% of Canadians with chronic back problems, and about 10% of those with headaches or fibromyalgia reported receiving regular health care from a chiropractor. Canadians with other chronic conditions as measured in the CCHS, including asthma, diabetes, mood disorders and anxiety also reported utilizing chiropractic care (Table 5).

## Discussion

Our study updates previous work and provides new evidence of the prevalence and characteristics of Canadians seeking chiropractic care between 2001 and 2010 and 2015 and 2018. We found a small increase in the national annual prevalence of Canadians consulting a chiropractor in the previous 12 months that ranged from 11.0% in 2001 to 11.4% in 2010, and in those reporting receiving regular health care from a chiropractor from 7.5% in 2015 to 7.9% in 2018. The prevalence of utilization varied by province and was found to be highest in provinces west of Ontario and lowest in the Atlantic provinces. The age-specific prevalence of those seeking chiropractic services was highest in ages 35–49 years and age specific prevalences remained stable over time, except for a small increase in those 65–79 years old. A higher percentage of Canadians identifying as white, Canadian-born, in the highest quintile of household income, overweight, physically active and in excellent health reported seeking chiropractic services. The most common reported chronic conditions (as measured in the CCHS) among Canadians seeking chiropractic care were chronic back problems, fibromyalgia, headaches, and arthritis.

Our study adds to the few Canadian studies that have reported national trends in the prevalence of utilization of chiropractic services over time. We found a small increasing trend in Canadians reporting consulting a chiropractor in the preceding 12 months between 2001 and 2010, and for those reporting receiving regular health care from a chiropractor from 2015 to 2018. These findings are the same as those reported most recently by Wong et al. who also used CCHS data [19], similar to those of Canizares et al. who reported an increase in surveyed Canadians consulting a chiropractor from 10.7% in 1994-95 to 13.4% in 2010-11 [18], and to the estimated linear increase in utilization in Canada reported by Beliveau et al. [16]. The reported differences between studies are likely related to different methodological approaches and data availability; for example, Canizares et al. used the longitudinal component of the Canadian National Population Health Survey [18], while Beliveau et al. conducted a scoping review of global studies [16]. Further, these studies reported average utilization trends at the national level, but there is a paucity of studies reporting the prevalence of utilization of chiropractic services at the provincial level.

Most provincial level studies have used cross-sectional prevalence data at one time point only [24–26], with only one reporting the trend in utilization at a single province level [24] and another across provinces but limited to chronic back problems [19]. Our study fills this gap by reporting the trend in prevalence of utilization of chiropractic services for each province and territories over time. In general, the trend in utilization has remained relatively stable or underwent minimal changes between 2001 and 2010 and 2015 and 2018, with notable higher values reported in Ontario and the Western provinces. The higher prevalence of utilization in these provinces may be attributed to several factors, including their early regulatory legislation as evidence of the profession’s organization, and having had or continuing to have some level of public health care funding for chiropractic services (see Table 6). However, the nature and amount of such funding has changed over time, with the governments in Saskatchewan and Ontario having delisted chiropractic services, British Columbia and Alberta limiting funding to certain populations, and only Manitoba continuing to provide funding to the general population albeit for limited number of visits. Despite this change in public funding, the prevalence of utilization in these provinces remained relatively stable with only Saskatchewan showing an increasing trend between 2001 and 2010. Stabile and Ward also found no effect of delisting on the overall use of chiropractic services, but we were unable to confirm their finding of a potential negative effect on the number of services demanded [27]. This apparent lack of effect of delisting may be the result of shifting the provision of care and related costs towards those with higher incomes, with proactive behaviours towards health decisions and services, having available resources to access health services, and/or being more likely to seek care [17, 24, 27, 28]. In line with these potential explanations, our findings suggest that a higher proportion of Canadians with post-secondary education, incomes in 3rd to 5th quintile, employed, active and in very good perceived general health seek chiropractic services.

**Table 6:** A review and summary of provincial regulatory legislation and public funding of chiropractic services in Canada.^1^

Province/Territory	Regulatory Legislation^2^	Initiated Coverage	Changes in Coverage^3^	Delisting of Services	Current status in 2024
British Columbia	1934	1965:		2001	2002: Limited coverage*
Alberta	1923	1969: $200/yr	1994: $300/year 1995: $200/year	2009	2012: Seniors-$200/year
Saskatchewan	1943	1973: no limit	2010: delisted for all residents except those on low-income program: 12 visits/yr	2017	No change
Manitoba**	1945	1969: no visit limit	1989: 15 visits/yr 2002: 12 visits/yr 2018: 7 visits/yr	NA	No change: 7 visits/yr
Ontario^4^	1925	1970: $125/yr	1989: $220/yr 1994: $200/yr 1998: $150/yr	2004	No change
Quebec	1973	N/A			No change
New Brunswick	1958	N/A			No change
Nova Scotia	1974	N/A			No change
Prince Edward Island	1968	N/A			No change
Newfoundland	1992	N/A			No change
Territories***	1955 (Yukon)	N/A			No change

^1^. Adapted from Stabile and Ward, 2006. ^2^. Sunderland, 1993. ^3^. Annual coverage limits per year, with varying visits costs usually supplemented by patient co-play. ^4^. Brown, 2013

*British Columbia limited coverage to combine total of 10 visits for chiropractic, massage therapy, physical therapy or non-surgical podiatric visits

**Manitoba coverage continues, changes have seen decrease in number of visits but incremental year-to-year increases per visit as per government agreement

***Regulatory legislation has not been enacted in Northwest Territories and Nunavut (Boucher, 2016)

Furthermore, this shifting of the provision of cost transfers the economic burden to patients, potentially impacting accessibility of health care services or increasing the risk that patients will forgo treatments, particularly those with lower income and chronic conditions [17, 29]. Such differences in utilization of chiropractic services have recently been reported in a novel Danish study that demonstrated income and employment related social inequity impacted utilization beyond differences in health status [30]. Reflecting these potential explanations, our findings highlight lower prevalence of chiropractic utilization among Canadians with less than secondary school education, lowest quintile of household income, landed immigrant status, under weight, poor perceived quality of health, and who are 80 years and older.

It is estimated that in Canada, 22% of the population will be 65 years and older by 2042, and globally there will be a threefold increase in the population 80 years and older [31]. Older age has been associated with worse health, greater use of healthcare and related costs [18], and those with chronic LBP are less likely to consult a chiropractor or physiotherapist, but more likely to consult a medical doctor or nurse [19]. Our study supports previous findings of higher prevalence of Canadians consulting a chiropractor or receiving regular health care from a chiropractor between 35 and 49 years of age and lower prevalence amongst those 80 years and older, with trends remaining relatively stable over time for all age groups, except those between 65 and 79 years where a small increasing trend was found [18, 19]. The small increase in utilization over time by those between 65 and 79 years of age may reflect a cohort effect as noted by Canizares et al. [18] regarding complementary and alternative medicine use in general, i.e., those in later birth cohorts were more likely to utilize chiropractic services at older ages than those in earlier birth cohorts. However, the small and increasing trend in the prevalence of utilization of chiropractic services observed in Canadians between 65 and 79 years, combined with the growing health challenges faced by an increasing ageing population, requires further research to better understand the reasons for the lower prevalence of older adults seeking chiropractic services, and exploration of whether this is because of unmet demand, affordability or uncertainty of the role that chiropractors have in managing older adults [24]. 

We identified a higher percentage of Canadians identifying as white, non-immigrant (Canadian born) and having immigrated more than 10 years earlier consulted or received regular health care from a chiropractor. Recent surveys of Canadian chiropractors reported that 80% of chiropractors identified as Caucasian and 70% as having Canadian ancestry [32], with almost 80% who reported observing important cultural health disparities in the health care system and identifying cost and language as barriers to people seeking chiropractic care [33]. With a Canadian population that is increasingly diverse [34], further research is needed to understand how cultural and racial factors may impact chiropractic care seeking behaviours or access to services.

### Implications

Our findings have implications for professional leadership, policy makers, and health planning in Canada. They provide an up-to-date analysis of Canadians who access chiropractic services and identify associated factors and inequities to such access over time. Given the growing burden of musculoskeletal conditions, particularly LBP [6], the significant unmet need for rehabilitation services [3], crisis in primary health care delivery and health workforce [9, 10], and being the second most accessed health provider for chronic low back pain [19], our findings could assist policy-makers from countries like Canada in informing potential strategies to address such notable challenges and gaps. Due to the survey design, populations such as those living on reserve and other Aboriginal settlements were excluded, thus we were unable to assess their utilization of chiropractic services. Further research focussing on addressing inequities to accessing chiropractic care and impact on health outcomes is needed. Future policy relevant research focusing on the needs of Canadians by addressing public access to care for the most disadvantaged and diverse communities, including access to chiropractors in health care teams is warranted.

### Strengths and limitations

Strengths of our study included the use of large, comprehensive, and representative population-based representing about 98% of community dwelling Canadians aged 12 years or older. To our knowledge, this is the first study of assessing chiropractic utilization trend over time, on both national level and characteristic-stratified level. We stratified the prevalence estimate by a wide range of biopsychosocial factors, providing fundamental insights for future studies on association analysis. There are limitations, such as potential measurement error with self-reported data and the study’s timeframe not fully capturing the current healthcare needs. The validity and reliability of certain questions in the CCHS (e.g., utilization of chiropractic services) are unknown, but have been used in previous studies exploring utilization of health services [12, 17, 19]. In addition, CCHS sampling frame includes individuals living in private dwellings only, and results may not be generalizable to other populations (e.g., persons living in institutions, on reserve and other First Nations settlements).

## Conclusions

The national annual prevalence of utilization of chiropractic services by Canadians slightly increased over time but varied by province and respondents’ socioeconomic and health characteristics. Chronic back problems were the most common reported chronic condition among those seeking chiropractic care. This up-to-date and comprehensive population-based study on chiropractic utilization in Canada can be used to inform decision-making concerning health human resources and access to rehabilitation care for different musculoskeletal disorders, particularly those in disadvantaged and diverse communities.

### Declaration Section

## Data Availability

The data that support the findings of this study are available from Statistics Canada, but restrictions apply to the availability of these data, which were accessed remotely by submitting SAS programs to Statistics Canada and receiving results outputs following a confidentiality review for the current study. As a result, the data are not publicly available. However, they can be obtained from Statistics Canada upon reasonable request.
